# OsPUP1 Modulates Cytokinin Distribution and Antioxidant Defense to Regulate Heat Stress Tolerance in Rice

**DOI:** 10.3390/antiox15070799

**Published:** 2026-06-26

**Authors:** Shujie Wang, Qiang Yu, Junwen Zhang, Yingfeng Wang, Guilian Zhang, Huabing Deng, Xiong Liu, Xuedan Lu, Qiuhong Chen, Feng Wang, Wenbang Tang, Yunhua Xiao

**Affiliations:** 1 Hunan Provincial Key Laboratory of Rice and Rapeseed Breeding for Disease Resistance, College of Agronomy, Hunan Agricultural University, Changsha 410128, China; 2 Yuelushan Laboratory, Changsha 410128, China; 3 State Key Laboratory of Hybrid Rice, Hunan Hybrid Rice Research Center, Changsha 410125, China

**Keywords:** rice, cytokinin, purine permease, *OsPUP1*, heat stress

## Abstract

Heat stress has emerged as one of the major environmental factors constraining rice growth. In our previous studies, OsPUP1, a member of the purine transporter protein family in rice, has been verified to be involved in the transport of cytokinin (CK). Unfortunately, the role of the *OsPUP1* gene in the heat stress response has not been fully elucidated to date. In this study, we show that overexpression of *OsPUP1*, encoding a purine permease in rice, leads to severe growth inhibition and oxidative damage under heat stress. *OsPUP1* overexpression lines exhibited increased accumulation of peroxides and malondialdehyde (MDA), along with significantly reduced activities of superoxide dismutase (SOD) and peroxidase (POD). Together with previous findings, these results suggest that OsPUP1 modulates CK transport, thereby influencing the spatial distribution of CK within the plant, which in turn regulates antioxidant defense mechanisms and heat stress response. This study uncovers a key role for *OsPUP1* in controlling thermotolerance in rice and highlights the importance of CK distribution in plant adaptation to heat stress.

## 1. Introduction

Global warming has had a profound impact on human production and life [1]. In recent years, the frequent occurrence of extreme heat events has posed a serious threat to global grain production [2]. Studies show that for every 1 °C increase in global average temperature, the yields of major food crops decline by approximately 3–8%, with rice production decreasing by 3.2% [3]. As a globally significant staple crop, rice serves as the primary food source for about 50% of the world’s population [4]. During the flowering period of rice, heat stress affects the cracking of anthers and the fertility of pollen [5], and during the grain-filling period, heat stress reduces grain weight, thereby affecting the seed setting rate, with variations observed among different cultivars [6]. Therefore, heat stress has already posed a serious threat to the growth and development of rice and its grain production.

Heat stress induces substantial physiological and biochemical impairments in plants, directly interfering with their metabolic processes and gene expression patterns, which consequently disrupts crop growth and development. At the physiological and biochemical level, thermal stress triggers oxidative stress in plants, damages photosystem II, and compromises normal photosynthetic and respiratory functions, leading to the overproduction of reactive oxygen species (ROS) [7]. The accumulation of ROS further causes oxidative damage to membranes, organelles, and proteins, while also impairing membrane lipid permeability [8]. As one of the signaling molecules in heat stress, ROS is primarily generated in chloroplasts, mitochondria, and peroxisomes. When ROS accumulation causes cellular damage, the antioxidant system becomes activated to eliminate excess ROS and maintain cellular homeostasis [9].

Plant hormones regulate multiple critical processes in rice growth and development, including growth, nutrient response, and biotic/abiotic stress responses [10]. Recent studies have demonstrated that plant hormones play a pivotal role in plant adaptation to environmental stressors [11]. Under heat stress, abscisic acid rapidly accumulates in plants [12], enhances antioxidant enzyme activity, and mitigates the accumulation of ROS [13]. Similarly, brassinosteroids alleviate heat-induced damage by increasing antioxidant compounds [14]. Foliar application of CK and brassinosteroids has been demonstrated to mitigate heat stress-induced damage in rice plants [15]. Appropriate application of exogenous kinetin (KT), a synthetic CK with high biological activity, enhances thermotolerance in rice seedlings by alleviating membrane damage and reducing ROS accumulation [16]. Furthermore, alternate wetting and drying irrigation can augment heat tolerance in rice by increasing CK levels in panicles [17].

As a crucial class of phytohormones, CKs participate in multiple physiological processes including stem cell division, panicle development, plant architecture establishment, nitrogen utilization efficiency, and environmental adaptation [18]. To exert their functions, CKs are typically transported to target tissues [19] via four protein families: purine permeases (PUP) [20,21,22], ATP-binding cassette transporters (ABC) [23,24], azaguanine transporters (AZG) [25,26], and equilibrate nucleoside transporters (ENT) [27,28]. Given the differences in their protein subcellular localization, the four cytokinin transporters exhibit distinct functional specializations: ABC transporters (e.g., AtABCG14) are localized to the plasma membrane and mediate the long-distance bidirectional transport of CK via an efflux mechanism. The PUP family is widely distributed across multiple subcellular compartments, including the plasma membrane, vacuole, and endoplasmic reticulum, and is involved in the regulation of local CK homeostasis. AZG proteins (e.g., AZG2) localize to the plasma membrane and endoplasmic reticulum and also mediate the influx of free-base CKs; their unique functions include mediating the crosstalk between CK and auxin signaling, as well as regulating lateral root development and the response to salt stress. The ENT family specifically recognizes CKs in the riboside form and is responsible for the reabsorption of CKs in roots [29]. As receptors of cytokinin signals, response regulators (RRs) directly participate in the transcription of related genes in the nucleus and precisely regulate the balance of cytokinin signals within the plant [30].

In rice, a total of 12 *PUP* family members have been identified [20]. Previous studies have demonstrated that OsPUP7 participates in the transport of CK derivatives, influencing plant growth, developmental processes, and stress responses [20,21]. As a homolog of OsPUP7, OsPUP4 is responsible for loading CK into the vascular bundles to regulate long-distance CK transport, thereby maintaining CK homeostasis in plants [21]. Earlier research has shown that OsPUP1 localizes to the endoplasmic reticulum and is primarily expressed in root vascular tissues, with its expression being induced by CK treatment. In the *OsPUP1*-overexpressing plants, the CK content decreased in the aerial parts while increasing in the root system. We preliminarily propose that OsPUP1 functions as a CK transporter responsible for unloading CKs from the vascular system via cell-to-cell transport [22]. This study aims to elucidate the regulatory role of *OsPUP1* in thermotolerance of rice under heat stress, thereby providing a theoretical foundation for further clarifying the relationship between CK and heat stress tolerance in rice.

## 2. Materials and Methods

### 2.1. Plant Materials and Growth Conditions

The *japonica* cultivar Zhonghua 11 (ZH11) was employed as the wild-type control, with two *OsPUP1* overexpression lines (OE1 and OE2) used as experimental materials. The transgenic seeds were provided by Prof. Xiao from Hunan Agricultural University, Changsha, China. All plants were cultivated in a controlled environment chamber maintained at 28 °C with 70% relative humidity, under a 12 h light/12 h dark photoperiod and a light intensity of 30,000 lux. Hydroponic growth was sustained using Kimura B nutrient solution.

### 2.2. Heat Stress Treatment

First, 8-day-old seedlings initially grown under normal conditions were subjected to heat stress in an illuminated incubator at 45 °C for 36 h, with 70% relative humidity, under a 12 h light/12 h dark photoperiod and a light intensity of 30,000 lux [31]. Following the stress treatment, the seedlings were transferred back to a 28 °C chamber for a 7-day recovery period, while all other environmental parameters remained unchanged. Plant phenotypes were photographed both pre-treatment and post-recovery, and survival rates were subsequently analyzed. Thermometers were placed at different positions within the incubator, and the experimental materials were placed into the incubator for treatment only after all thermometers stabilized at 45 °C. After the completion of the treatment, the survival rate of the seedlings was counted. The survival criterion for seedlings after treatment recovery was defined as follows: 7 days after the recovery period, seedlings with green stems and leaves as well as newly grown roots are regarded as surviving individuals. Survival rate = (number of surviving seedlings/total number of seedlings before treatment) × 100%.

### 2.3. Effect of Exogenous Kinetin Application on Heat Stress in OsPUP1 Overexpression Lines

First, 10-day-old OE2 seedlings with uniform growth were selected for exogenous KT treatment at concentrations of 10^−9^ M. The root systems were immersed in Kimura B nutrient solution containing corresponding KT concentrations, while the second leaves received foliar application of KT at equivalent concentrations. All treated seedlings were subsequently transferred to a 45 °C illuminated incubator for heat stress exposure. When distinct phenotypic variations became evident, all plant materials were moved to a 28 °C normal-temperature illuminated incubator for a 7-day recovery period prior to analytical assessment and photographic documentation.

### 2.4. RNA Extraction and qRT-PCR Analysis

Post 36 h heat stress, samples of wild-type plants (WT), OE1, and OE2 were placed in 2.0 mL centrifuge tubes prefilled with sterilized steel beads, rapidly frozen in liquid nitrogen, and ground into a fine powder. Total RNA was subsequently extracted using the TRIzol method. cDNA synthesis was performed following the manufacturer’s protocol with the HiScript II Q Select RT SuperMix for qRT-PCR (+gDNA wiper) kit (R223-01, Vazyme, Nanjing, China). qRT-PCR assays were conducted using the rice *UBIQUITIN* gene as an internal reference, with ChamQ Universal SYBR qPCR Master Mix (Q711-02, Vazyme, Nanjing, China) on a CFX96 Real-Time Detection System (1855195, Bio-Rad Laboratories, Hercules, CA, USA). Relative gene expression levels were quantitatively analyzed using the Comparative Ct method across three biological replicates. The primer sequences used for qRT-PCR are shown in Supplemental Table S1.

### 2.5. NBT/DAB Staining

The accumulation of peroxides in leaves was detected via DAB/NBT staining [32]. Nitroblue tetrazolium (NBT) reacts with superoxide anions (O_2_^•−^) to form an insoluble blue formazan precipitate. Based on this principle, an NBT solution (0.1%, pH 7.8) is employed for the detection of superoxide anions in plant tissues, typically using leaf samples. Diaminobenzidine (DAB) serves as a substrate for peroxidase (POD), and enzymatic activity results in the formation of a brown precipitate at reactive sites. For staining, 10 mg each of NBT (CN7731, Coolaber, Beijing, China) and DAB (CD4181, Coolaber, Beijing, China) powders were separately dissolved in 10 mL of distilled deionized water (ddH_2_O) to prepare 0.1% staining solutions. Leaf tips from WT, OE1, and OE2 samples subjected to 36 h of heat stress, along with untreated control samples, were excised to fit 2 mL centrifuge tubes. The samples were immersed in 2 mL of NBT or DAB staining solution, respectively, and incubated at 37 °C in aluminum foil-wrapped tubes for 2 days. After staining, samples were decolorized by heating in 75% ethanol for 10 min, followed by preservation in fresh 75% ethanol for imaging.

### 2.6. Determination of Physiological Indices

First, 8-day-old seedlings of WT, OE1, and OE2 lines were subjected to heat stress treatment, with control groups maintained at 28 °C in an incubator. After 36 h of treatment, leaves from identical positions were collected and immediately frozen in liquid nitrogen using 2 mL EP tubes, and then ground into powder for physiological parameter assessments. Each sample (0.1 g aliquot) was analyzed using Solarbio assay kits (Solarbio, Beijing, China) to determine SOD activity, POD activity, and MDA content, and three biological replicates were performed.

SOD activity was quantified by measuring the absorbance at 450 nm of water-soluble yellow formazan generated from the reaction between O_2_^•−^ and WST-1 (BC5165, Solarbio, Beijing, China) [33,34]. POD activity was evaluated based on the characteristic absorbance at 470 nm from H_2_O_2_ oxidation substrates (BC0090, Solarbio, Beijing, China) [35]. Lipid oxidation levels were monitored via MDA quantification. Under acidic and heat conditions, MDA condenses with thiobarbituric acid (TBA) to form a brown-red trimethine compound (3,5,5-trimethyloxazolidine-2,4-dione), exhibiting maximum absorption at 532 nm (BC0025, Solarbio, Beijing, China) [36]. Absorbance values were measured at respective wavelengths and analyzed accordingly. All experimental steps and procedures using the kits were performed strictly following the manufacturer’s protocols.

### 2.7. Statistical Analysis

Data are presented as mean ± SD, and statistical analysis was performed using Microsoft Excel 2019 (version 16.0). Student’s *t*-test was employed to compare the means between two groups, and one-way analysis of variance (ANOVA) was applied to compare differences among multiple groups. Graphs were plotted using GraphPad Prism (version 8.01).

### 2.8. Accession Numbers

Sequence data from this article can be found in the Rice Genome Annotation Project (http://rice.plantbiology.msu.edu/ accessed on 11 May 2026) under the following accession numbers: *OsPUP1* (*LOC_Os03g08880*), *OsRR1* (*LOC_Os04g36070*), *OsRR2* (*LOC_Os02g35180*), *OsRR4* (*LOC_Os01g72330*), *Ubiqutin2* (*LOC_Os02g06640*), *OsFe^+^-SOD* (*LOC_Os06g05110*), *OsAPX1* (*LOC_Os03g17690*), *OsCATB* (*LOC_Os06g51150*), *OsHSP70* (*LOC_Os11g47760*), and *OsHSP90* (*LOC_Os06g50300*).

## 3. Result

### 3.1. OsPUP1 Negatively Regulates Heat Stress Tolerance in Rice Seedling

**Figure 1:**
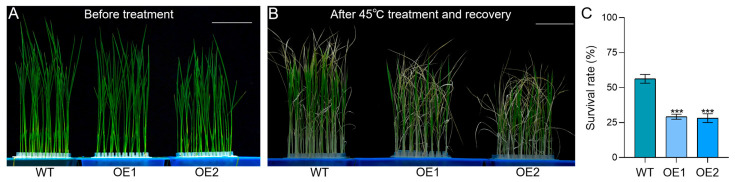
Phenotypes of WT, OE1, and OE2 seedlings under heat stress. (**A**) Seedlings were hydroponically at 28 °C for 8 d. Scale bars = 5 cm. (**B**) The phenotypes of WT, OE1, and OE2 seedlings after heat stress at 45 °C for 36 h followed by normal temperature recovery at 28 °C for 7 d. Scale bars = 5 cm. (**C**) Survival rates of WT, OE1, and OE2 seedlings as depicted in (**B**). *n* = 3, bar = SD, *** *p* ≤ 0.001 in Student’s *t*-test.

To investigate whether *OsPUP1* regulates HS tolerance, 8-day-old seedlings of WT and two *OsPUP1*-overexpressing lines (OE1 and OE2) were subjected to heat stress for 36 h, followed by a 7-day recovery period under normal growth conditions (Figure 1A,B). Statistical analysis of post-recovery survival rates revealed that OE1 and OE2 exhibited significantly lower survival rates compared to the WT (Figure 1C). These results indicate that *OsPUP1* overexpression significantly reduces thermotolerance during the seedling stage in rice.

### 3.2. Exogenous Application of Kinetin Partially Restores Thermotolerance in OsPUP1-Overexpressing Plants

**Figure 2:**
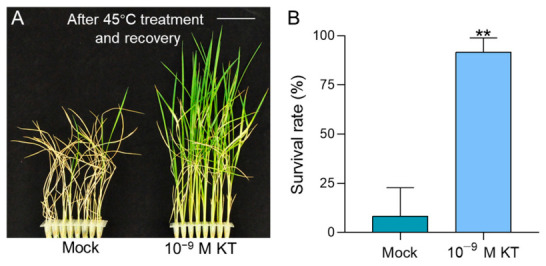
The effect of the application of exogenous Kinetin on heat stress of *OsPUP1*-OE2. (**A**) Phenotypes of heat treatment recovery in 10^−9^ M KT treatment and untreated control groups. Scale bars = 5 cm. (**B**) Survival rates of OE2 as depicted in (**A**). *n* = 3, bar = SD, ** *p* ≤ 0.01 in Student’s *t*-test.

Compared to the WT, the shoot of *OsPUP1*-overexpressing seedlings exhibited reduced CK content [22]. To further investigate whether the decreased CK content in the shoot of *OsPUP1*-overexpressing seedlings contributes to their reduced thermotolerance, we administered an exogenous application of 10^−9^ M KT to both the roots and leaves of uniformly grown OE2 seedlings, followed by heat stress (Figure 2A). Survival rate analysis after recovery revealed a significant increase in the survival of KT-treated OE2 seedlings compared to the control group without KT application (Figure 2B). These results further indicate that exogenous KT application can partially restore the thermotolerance in overexpression lines, with the reduced CK content in aerial parts potentially contributing to the diminished heat resistance observed in *OsPUP1*-overexpressing plants.

### 3.3. Reactive Oxygen Species Accumulate Significantly in OsPUP1 Overexpression Lines Under Heat Stress

**Figure 3:**
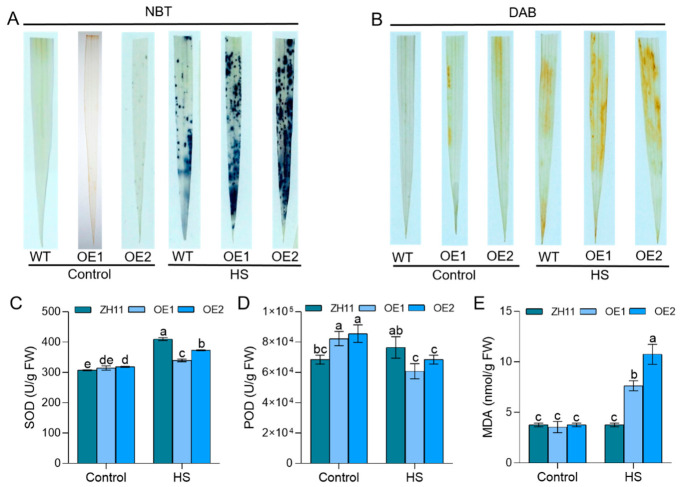
Reactive oxygen species in 8-day-old seedlings of WT and *OsPUP1* overexpression lines under heat stress. (**A**) NBT staining of seedling leaves in WT, OE1, and OE2 lines under control conditions and after heat stress. (**B**) DAB staining of seedling leaves in WT, OE1, and OE2 lines under control conditions and after heat stress (**C**) Determination of SOD activity in WT, OE1, and OE2 seedlings after 36 h of 45 °C treatment. (**D**) Determination of POD activity in WT, OE1, and OE2 seedlings after 36 h of 45 °C treatment. (**E**) Measurement of MDA content in WT, OE1, and OE2 seedlings after 36 h of 45 °C treatment. Data are expressed as the mean ± SD. Different letters indicate significant differences by one-way ANOVA test (*n* = 3, *p* ≤ 0.05).

Heat stress induces the accumulation of ROS in plants, and ROS burst can lead to oxidative damage in plant cells [37]. To investigate whether *OsPUP1* overexpression exacerbates heat-induced oxidative stress, we measured the accumulation of H_2_O_2_ and O_2_^•−^ in WT and overexpression lines under heat stress conditions. Eight-day-old seedlings of WT and *OsPUP1*-overexpressing lines were subjected to 45 °C for 36 h, followed by immediate staining with DAB and NBT. Under control conditions, no significant difference in leaf staining was observed between *OsPUP1*-overexpressing and WT plants. However, under heat stress, the overexpression lines exhibited noticeably deeper staining (Figure 3A,B). These results indicate that *OsPUP1*-overexpressing plants accumulate higher levels of H_2_O_2_ and O_2_^•−^ under heat stress.

To quantify the abnormal accumulation of ROS in the overexpression lines, we measured several physiological indicators [38], including the activities of SOD, POD, as well as the MDA content—a marker of lipid peroxidation—in both WT and *OsPUP1*-overexpressing plants under heat stress. After 36 h of heat stress, SOD and POD activities decreased rapidly in OE1 and OE2, whereas they increased significantly in WT plants (Figure 3C,D). Concurrently, MDA accumulation was markedly elevated in the OE lines (Figure 3E). These findings demonstrate that the overexpression of *OsPUP1* suppresses the heat stress-induced activation of antioxidant enzymes (SOD and POD), leading to uncontrolled ROS accumulation and aggravated membrane lipid peroxidation (reflected by MDA buildup), reducing tolerance to heat stress.

### 3.4. Transcription Levels of Antioxidant Enzyme Genes Were Significantly Reduced in the OsPUP1-Overexpressing Line Under Heat Stress

**Figure 4:**
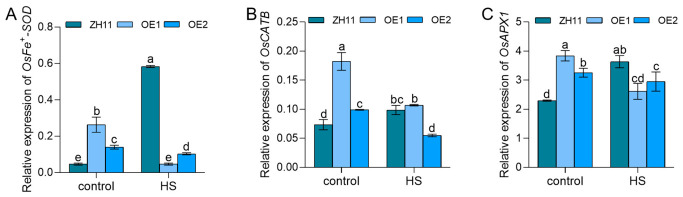
Relative expression levels of antioxidant enzyme genes in heat-stressed and control groups. (**A**) Relative *OsFe^+^-SOD* expression in the plants tested by qRT-PCR. *UBIQUITIN2* gene was used as an internal reference. (**B**) Relative *OsCATB* expression in the plants tested by qRT-PCR. The *UBIQUITIN2* gene was used as an internal reference. (**C**) Relative *OsAPX1* expression in the plants tested by qRT-PCR. The *UBIQUITIN2* gene was used as an internal reference. Data are expressed as the mean ± SD. Different letters indicate significant differences by one-way ANOVA test (*n* = 3, *p* ≤ 0.05).

To elucidate the molecular mechanism underlying the alterations in antioxidant enzyme activities described above, we further examined the relative expression levels of three antioxidant enzyme genes (*OsFe^+^-SOD*, *OsCATB*, and *OsAPX1*) [39,40,41] following 36 h of heat stress. The results indicated that, under heat stress, the transcript levels of *OsFe^+^-SOD* and *OsAPX1* in both OE1 and OE2 were significantly lower than those in the control group (Figure 4A,C). After heat stress, the transcript level of *OsCATB* in OE1 and OE2 decreased significantly compared to the untreated condition, whereas it increased markedly in the WT plants (Figure 4B). These findings demonstrate that the overexpression of *OsPUP1* negatively regulates the transcription of antioxidant enzyme genes, thereby impairing the ROS-scavenging capacity of the plants and consequently reducing their thermotolerance.

### 3.5. Differential Regulation of Heat Stress Defense Genes at Transcriptional Level

**Figure 5:**
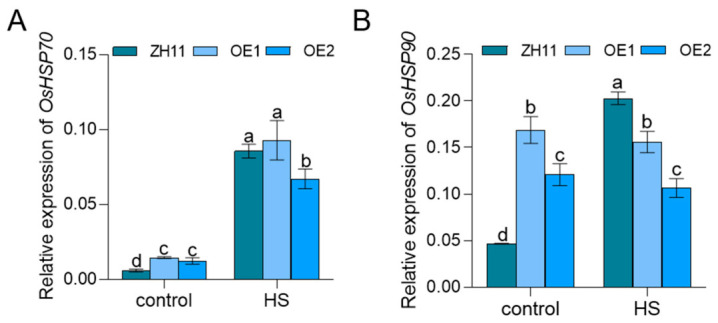
Relative expression levels of stress defense genes in heat-stressed and control groups. (**A**) Relative *OsHSP70* expression in the plants tested by qRT-PCR. The *UBIQUITIN2* gene was used as an internal reference. (**B**) Relative *OsHSP90* expression in the plants tested by qRT-PCR. The *UBIQUITIN2* gene was used as an internal reference. Data are expressed as the mean ± SD. Different letters indicate significant differences by one-way ANOVA test (*n* = 3, *p* ≤ 0.05).

Heat shock proteins (HSPs), primarily functioning as molecular chaperones regulating protein folding and unfolding, are typical thermal response proteins [9]. To investigate the role of *OsPUP1* in plant heat stress response, the expression levels of *OsHSP70* and *OsHSP90* in WT, OE1, and OE2, under heat stress were detected using quantitative real-time PCR (qRT-PCR). Following heat stress, *HSP70* expression was significantly upregulated in both overexpression lines (Figure 5A). However, the response of *HSP90* exhibited marked heterogeneity. While its expression was notably induced in WT, no significant alterations were observed in the overexpression lines (Figure 5B). These findings suggest that *OsPUP1* may influence plant thermotolerance through the modulation of *HSP* transcription.

### 3.6. Transcription Levels of RRs Were Significantly Reduced in the OsPUP1-Overexpressing Line Under Heat Stress

**Figure 6:**
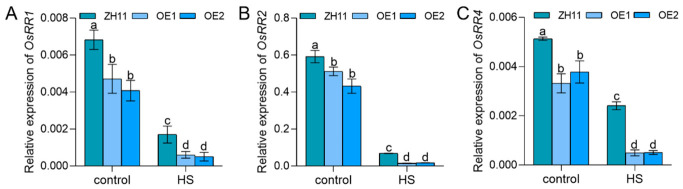
Relative expression levels of *RRs* in heat-stressed and control groups. (**A**) Relative *RR1* expression in the plants tested by qRT-PCR. The *UBIQUITIN2* gene was used as an internal reference. (**B**) Relative *RR2* expression in the plants tested by qRT-PCR. The *UBIQUITIN2* gene was used as an internal reference. (**C**) Relative *RR4* expression in the plants tested by qRT-PCR. The *UBIQUITIN2* gene was used as an internal reference. Data are expressed as the mean ± SD. Different letters indicate significant differences by one-way ANOVA test (*n* = 3, *p* ≤ 0.05).

The transcriptional response to cytokinins is predominantly mediated by the RR family [42,43], which can serve as markers for identifying cytokinin-responsive gene transcription [44]. To investigate whether *OsPUP1* overexpression suppresses cytokinin signaling and thus reduces heat tolerance, we analyzed the expression levels of *RRs* in WT, OE1, and OE2 under both normal and heat stress conditions. Under ambient temperature, *RR* expression in the overexpression lines was significantly lower than in WT. Following heat stress, *RR* expression decreased in both WT and overexpression lines, with a more pronounced reduction observed in the overexpression lines, resulting in the lowest expression levels across all treatments (Figure 6A–C). These findings indicate that *OsPUP1* overexpression leads to decreased transcription of *RRs*, attenuating cytokinin signaling and consequently compromising the plant’s heat tolerance.

## 4. Discussion

Rice, as the primary staple food for more than half of the global population, faces significant challenges to its safe production due to the increasing frequency of extreme climatic events. Among these challenges, heat stress has emerged as a critical environmental constraint limiting rice yield [45]. CK plays a crucial role in plant abiotic stress resistance. Studies have demonstrated that drought and saline-alkali tolerance significantly influence endogenous CK levels in plants, while osmotic stress-dependent hydropatterning is also associated with the distribution of CK signaling [46]. Exogenous application of an appropriate concentration of CK onto rice leaves mitigates heat stress-induced damage, thereby enhancing thermotolerance in rice plants [47]. Similarly, foliar spraying with synthetic cytokinin KT at suitable levels improves the heat tolerance of rice seedlings by modulating the dynamic equilibrium of ROS within the plant system [16]. In contrast, the role of endogenous CK transport in regulating plant thermotolerance remains poorly documented. Overexpression of *OsPUP1* reduces CK content in the aerial parts of rice plants. Therefore, this study employed WT, OE1, and OE2 generated in the ZH11 background for heat stress. The experimental results show that under heat stress conditions, the survival rates of OE1 and OE2 are significantly lower than that of the wild type, indicating that overexpression of *OsPUP1* significantly reduces the thermotolerance of rice at the seedling stage. We therefore hypothesize that OE1 and OE2 weaken the heat stress tolerance of rice seedlings by reducing endogenous cytokinin levels. To verify this hypothesis, we exogenously applied an appropriate dosage of KT to wild-type (WT) and OE2 plants. The results show that exogenous KT can partially restore the thermotolerance of the overexpression lines, which further indicates that reduced cytokinin content in the aboveground part is a key factor leading to decreased thermotolerance in *OsPUP1*-overexpressing plants.

Elevated temperatures accelerate water loss, alter specific protein structures, promote ROS accumulation, damage cell membrane lipids, and disrupt plant cellular architecture, potentially leading to DNA mutations [48], ultimately resulting in plant wilting and mortality [49]. NBT and DAB staining further revealed elevated levels of H_2_O_2_ and O_2_^−^ in the two *OsPUP1*-overexpressing lines under heat stress compared to the control. Concurrently, heat stress suppresses the activity of antioxidant enzymes. Following heat exposure, SOD and POD activities in OE1 and OE2 lines decreased rapidly and were significantly lower than those in the control group. The decline in enzyme activity impedes thorough peroxide decomposition, leading to rapid ROS accumulation. Moreover, MDA accumulation in both OE1 and OE2 was markedly higher than in the WT, indicating that overexpression exacerbates membrane lipid peroxidation damage, disrupts cellular integrity, and consequently causes plant wilting and death. These findings align with previous research demonstrating that CK regulate thermotolerance by modulating ROS homeostasis in plants. In this experiment, reduced CK levels in OE1 and OE2 lines relative to the WT corresponded to diminished heat tolerance. Further physiological analyses revealed significant oxidative damage in OE1 and OE2, characterized by peroxide accumulation and increased MDA content. Subsequent examination of key antioxidant enzyme genes showed significant transcriptional repression of *Fe^+^-SOD*, *APX1*, and *CATB* [39,40,41], which directly corresponds to the observed reductions in SOD and POD activities. This confirms a defect in the transcriptional activation of the antioxidant system. We further hypothesize that the direct cause of decreased thermotolerance in *OsPUP1*-overexpressing plants is that the downregulated transcription of antioxidant-related enzyme genes reduces enzyme activity, which in turn leads to the accumulation of ROS and membrane lipid peroxidation.

As one of the most abundant heat shock proteins in eukaryotic cells, when heat stress occurs, partially denatured proteins compete with heat shock factor (HSF) for binding to HSP70, thereby releasing and activating HSF to induce the heat stress response, which further enhances the expression and accumulation of HSP70 [50]. In *Arabidopsis thaliana*, HSP90 controls the initiation of stomatal cell lineages by phosphorylating the mitogen-activated protein kinase cascade (MPK3 and MPK6) and couples this process with stomatal development to adapt to heat stress [51]. This study observed that under heat stress, *HSP70* and *HSP90* exhibited completely distinct expression patterns in *OsPUP1* overexpression lines: *HSP70* was significantly induced, while *HSP90* was normally upregulated in wild-type plants but showed no significant change in overexpression lines. These results indicate that *OsPUP1* overexpression can respond to *HSP70* at the initial stage of the heat stress response, but the transcription of its co-functional *HSP90* is restricted, suggesting that the *HSP90*-related thermotolerance regulatory pathway may be inhibited. Combined with the result that exogenous KT partially restored thermotolerance, we speculate that cytokinin signaling may be involved in regulating the function of certain heat stress-related proteins. Elucidation of this fine regulatory mechanism warrants further in-depth exploration in future studies.

Previous research has demonstrated that the exogenous application of CK at specific concentrations can enhance plant thermotolerance. However, the molecular mechanisms underlying CK transport remain poorly elucidated. In the present study, OsPUP1, a putative CK transporter in rice, leads to downregulation of *RR* expression and exerts a negative regulatory effect on CK transport. This deficiency impaired the full activation of the plant’s defense mechanisms, reduced the antioxidant capacity of rice seedlings, and consequently diminished their ability to scavenge ROS. Our findings elucidate the link between CK transport, redox homeostasis, and heat tolerance, underscoring the pivotal role of CK in plant stress adaptation. Nevertheless, the precise mechanism through which CK regulates ROS dynamics in plants warrants further investigation.

## 5. Conclusions

The results of this study indicate that *OsPUP1* overexpression significantly reduces plant thermotolerance. Under heat stress treatment, the significant decrease in antioxidant enzyme activity in the OE1 and OE2 lines led to increased accumulation of ROS and exacerbated lipid peroxidation-induced damage to cell membranes. Notably, exogenous application of 10^−9^ M KT partially restored the thermotolerant phenotype in the overexpression lines. At the same time, *OsPUP1* overexpression under heat stress treatment led to decreased transcriptional activity of *RRs*, which may have resulted in weakened cytokinin signaling. In conclusion, *OsPUP1* overexpression likely impairs the ROS-scavenging capacity of plants, thereby negatively regulating heat tolerance, whereas exogenous KT treatment can partially mitigate this adverse effect. This study provides experimental evidence that cytokinin transporter proteins influence plant thermotolerance.

## Data Availability

The original contributions presented in this study are included in the article/Supplementary Materials. Further inquiries can be directed to the corresponding authors.

## Supplementary Materials

The following supporting information can be downloaded at: https://www.mdpi.com/article/10.3390/antiox15070799/s1, Table S1: The list of RT-qPCR primer pairs.
