# Supplementary material for: OsPUP1 Modulates Cytokinin Distribution and Antioxidant Defense to Regulate Heat Stress Tolerance in Rice
Source: Antioxidants (Basel). 2026 Jun 26;15(7):799. doi: 10.3390/antiox15070799 (PMC13404339; doi:10.3390/antiox15070799)
Supplement: Supplementary file 1 [file antioxidants-15-00799-s001.zip › antioxidants-4339983-supplementary.pdf]

Supplementary Materials

Table S1. The list of RT-qPCR primer pairs.

**Name**	**Forward (5'-3')**	**Reverse (5'-3')**
Ubiquitin2	GAGCCTCTGTTCGTCAAGTA	ACTCGATGGTCCATTAAACC
OsCATB	GTTCGGTTCTCCACAGTCGT	CCCTCCATGTGCCTGTAGTT
OsAPX1	CCAAGGGTTCTGACCACCTA	CAGTTCGGAGAGCTTGAGGT
OsFe^{+}-SOD	CTTGATGCCCTGGAACCTTA	GCCAGACCCCAAAAGTGATA
OsHSP70	GCCAAGCGTCAAGCAGTGACCAA	GGTCATCAAAGCGCCGCCCTAT
OsHSP90	TTTGGGCGAAGGTGACACTGCTA	TGGCAATGGTCCCAAGGTTCTTAAT
OsRR1	AGGATCAGCAGATGCATGAATG	GAGACGCTGTACGTCCTTGCTT
OsRR2	CATGGTGATGAATGCATCC	TGCTGCCATTGGACCATCT
OsRR4	TGAAGCTGCAACAGCTCA	AGTGGAGGACAATCTTGG
